# The relationship between breastfeeding and weight status in a national sample of Australian children and adolescents

**DOI:** 10.1186/1471-2458-12-107

**Published:** 2012-02-07

**Authors:** Jane A Scott, Su Y Ng, Lynne Cobiac

**Affiliations:** 1 Department of Nutrition and Dietetics, School of Medicine, Flinders University, Adelaide, South Australia; 2 Physical Activity and Nutrition Observatory: Research And Monitoring Alliance (PANORAMA), Flinders University, Adelaide, Australia; 3 CSIRO Food and Nutritional Sciences, Kintore Ave, Adelaide, South Australia

## Abstract

**Background:**

Breastfeeding has been shown consistently in observational studies to be protective of overweight and obesity in later life. This study aimed to investigate the association between breastfeeding duration and weight status in a national sample of Australian children and adolescents.

**Methods:**

A secondary analysis of the 2007 Australian National Children's Nutrition and Physical Activity Survey data involving 2066, males and females aged 9 to 16 years from all Australian states and territories. The effect of breastfeeding duration on weight status was estimated using multivariate logistic regression analysis.

**Results:**

Compared to those who were never breastfed, children breastfed for ≥6 months were significantly less likely to be overweight (adjusted odds ratio: 0.64, 95%CI: 0.45, 0.91) or obese (adjusted odds ratio: 0.51, 95%CI: 0.29, 0.90) in later childhood, after adjustment for maternal characteristics (age, education and ethnicity) and children's age, gender, mean energy intake, level of moderate and vigorous physical activity, screen time and sleep duration.

**Conclusions:**

Breastfeeding for 6 or more months appears to be protective against later overweight and obesity in this population of Australian children. The beneficial short-term health outcomes of breastfeeding for the infant are well recognised and this study provides further observational evidence of a potential long-term health outcome and additional justification for the continued support and promotion of breastfeeding to six months and beyond.

## Background

Globally, the number of overweight and obese children has increased rapidly since the mid-1980s [1]. In Australia, the combined prevalence of overweight and obesity doubled and the prevalence of obesity trebled between 1985 and 1997 [2]. While the prevalence appears to have reached a plateau in recent years, it remains high with 22% of Australian boys and 24% of girls aged 2 to 16 years reported as being either overweight or obese in 2007 [3].

Breastfeeding has been shown consistently in a number of systematic reviews and meta-analyses to be protective of overweight and obesity in later life [4-6]. Although different criteria have been used in individual studies to define overweight and obesity, the results of the studies have been similar [5]. Most recently, Horta and colleagues [5] conducted a meta-analysis which included 39 estimates of the effect of breastfeeding on the prevalence of overweight or obesity in later childhood or adult life and concluded that breastfed individuals were 22% less likely to be considered as overweight or obese than individuals who had not been breastfed. Harder and colleagues [7] reported an inverse association between duration of breastfeeding and the risk of overweight, with each additional month of breastfeeding associated with a 4% decrease in the odds of being overweight in later life. To date, the Promotion of Breastfeeding Intervention Trial (PROBIT) [8] is the only (cluster) randomised controlled trial that has investigated the effect of breastfeeding on obesity in later childhood and this study failed to find a lower prevalence of obesity between the intervention and the control groups.

The evidence for a protective effect of breastfeeding on childhood overweight and obesity is derived primarily from observational studies, a common limitation of which is the inadequate adjustment for other factors identified as being associated with childhood overweight and obesity. For instance, early-life determinants of childhood overweight and obesity include socio-demographic and maternal factors [9,10], physical activity [11] and sedentary behaviours including screen time [12,13] and sleep duration [11,13]. The apparent protective effect of breastfeeding on overweight and obesity appears to be attenuated when more factors are adjusted for in the analyses [6].

To date, there have been few studies investigating the association of breastfeeding and overweight or obesity in Australian children and adolescents. The studies that do exist were based on single centre studies in either Brisbane [14] or Perth [15,16] and have not controlled for the current energy intake and physical activity of the children studied. The current study utilises the nation-wide data from the 2007 Australian National Children's Nutrition and Physical Activity Survey (NCNPAS) [3] to investigate the association between breastfeeding at infancy and weight status in later childhood.

## Methods

The 2007 Australian NCNPAS was conducted by the Commonwealth Scientific and Industrial Research Organisation (CSIRO) Preventive Health National Research Flagship and the University of South Australia (UniSA) [3]. The survey sample was selected firstly by postcode (stratified by state/territory and capital city/rest of state), and then Random Digit Dialling was used to identify households within the selected postcodes with children aged 2-16 years. Only one child per participating household was the designated "study child". Where there was more than one child aged 2-16 years in the household, a Kish method of child selection was used to ensure adequate representation of age and gender within the sample. Four age groups were selected (2-3 years, 4-8 years, 9-13 years and 14-16 years) with a target quota of 1000 children in each age group. The South Australian sample was supplemented by an additional 400 children to allow more detailed estimates for that State when analysed separately. Ethics approval was obtained from the NHMRC registered Ethics Committees of CSIRO and UniSA.

Data were collected via a face-to-face home visit computer-assisted personal interview (CAPI) and a subsequent computer-assisted telephone interview (CATI) conducted 7-21 days later, across school and non-school days. A total of 4,487 children completed both the CAPI and CATI. Anthropometric measurements of the study child (recalled birth weight, measured height and weight) were taken, and socio-demographic data as well as infant feeding practices (has child ever been breastfed and breastfeeding duration) collected from the primary care-giver during the CAPI. Daily energy intake was estimated from standardised, computer-based, 24-h recall of food, beverage and dietary supplement intakes collected during the CAPI and CATI. The daily energy intake used in this analysis is the average of the two 24 h recalls. Participants aged 9-16 years used the validated Multimedia Activity Recall for Children and Adolescents (MARCA) [17] to provide two consecutive days of physical activity and 'use of time' recall data at both the CAPI and CATI. Data from the MARCA were used to estimate average daily duration of moderate to vigorous physical activity (MVPA), total screen time (i.e. time spent watching television, operating a computer, playing video games and texting) and hours of sleep across the 4 days sampled. These data were not available for children aged 2 to 8 years. Further details of the survey methodology can be obtained from the 2007 Australian NCNPAS Users Guide, available at: http://www.health.gov.au/internet/main/publishing.nsf/Content/AC3F256C715674D5CA2574D60000237D/$File/user-guide-v2.pdf

For the purposes of collecting reliable breastfeeding data, only children whose biological mother was either the primary care-giver or partner of the primary care-giver were eligible for inclusion (n = 4304). In addition, only children aged 9-16 years were included (n = 2073) due to the intention to adjust for physical activity in the multivariate analysis. Of these 2066 children had complete data and formed the final sample.

The body mass index (BMI) of each subject was calculated (kg/m^2^) and dichotomous outcome variables (Yes or No) for overweight (equivalent to an adult BMI ≥ 25 kg/m^2^, so included both overweight and obese children) and obese (equivalent to an adult BMI ≥ 30 kg/m^2^) were created using the International Obesity Taskforce age and gender specific cut-offs [18]. The primary explanatory variable of interest was breastfeeding duration, categorised as 0 months, < 2 months, 2- < 4 months, 4- < 6 months and ≥6 months. In this analysis breastfeeding refers to any breastfeeding as we were unable to distinguish between exclusive and partial breastfeeding.

The mean daily duration of MVPA was reduced to a dichotomous explanatory variable (≥60 min/day = sufficient duration and < 60 min/day = insufficient duration) while the mean daily screen-time duration was reduced to ≥2 h/day (excessive duration) and < 2 h/day (non-excessive duration) in accordance with the recommendations of the National Physical Activity Guidelines [19]. Mean daily sleep duration was classified as < 8 h/day, 8-10 h/day and > 10 h/day.

Data were analysed using the Statistical Package for the Social Sciences (SPSS version 17, Chicago, IL, USA). The relationship between breastfeeding duration and weight status was investigated in a logistic regression model using categorised duration of breastfeeding as the primary explanatory variable. Other variables known to be associated with childhood obesity were sequentially added in blocks to determine the extent to which the effect of breastfeeding on overweight (dependent variable) was reduced when adjusted for these suspected confounders. In the second model, the base model was adjusted for study child's gender and age, in the third model maternal characteristics (age when child was born, highest level of education and country of birth) were added and finally in the fourth model the study child's average estimated daily energy intake (MJ), MVPA, total screen time and hours of sleep were added. A test for linear association (p for trend) between breastfeeding duration and overweight was performed by entering breastfeeding duration as a linear variable in each model. This process was repeated using obesity as the dependent variable. The Hosmer-Lemeshow test was applied and all models were shown to have reasonable goodness of fit.

## Results

Table 1 shows the characteristics of the 2066 subjects eligible for this analysis. The majority of the children (92.4%) had been breastfed and more than half (54.2%) were breastfed for 6 months or more. The mean age of mothers at the time the study child was born was 29.8 ± 5.3 years and more than half of the mothers (57.4%) had completed 12 or more years of schooling. The majority of children (69.6%) were in the healthy weight range for their age, while 18.7% were overweight and a further 6.4% were obese. The majority of children (78.1%) met the National Physical Activity Guidelines of 60 min or more of MVPA/day but failed (83.1%) to meet the screen time guidelines of less than 2 h of television or video and computer games.

**Table 1:** Characteristics of subjects (n = 2066)

Characteristics	N	%
Gender of study child		
Male	1010	48.9
Female	1056	51.1
Age group of study child		
9-13 years	1046	50.6
14-16 years	1020	49.4
Maternal age (years ± SD)	42.7 ± 5.6	
Maternal age at birth of study child (years ± SD)	29.8 ± 5.3	
Maternal highest primary/secondary education		
< Year 10	98	4.7
Year 10 and 11	782	37.9
Year 12 or equivalent	1186	57.4
Maternal birth country		
Australia/New Zealand	1645	79.6
United Kingdom/Ireland	178	8.6
Asia and Indian sub-continent	87	4.2
All other countries	156	7.6
Study child ever been breastfed	1908	92.4
Breastfeeding duration		
0 months	173	8.4
< 2 months	263	12.7
2-< 4 months	204	9.9
4-< 6 months	307	14.9
≥ 6 months	1119	54.2
Weight status^a ^of study child		
Underweight	108	5.3
Healthy weight	1438	69.6
Overweight	387	18.7
Obese	133	6.4
Average^b ^duration of moderate-to-vigorous physical activity		
< 60 minutes/day	452	21.9
≥ 60 minutes/day	1614	78.1
Average^b ^screen-time duration of study child		
< 2 hours/day	350	16.9
≥ 2 hours/day	1716	83.1
Average^b ^sleep duration of study child		
< 8 hours/day	90	4.4
8-10 hours/day	1045	50.6
> 10 hours/day	931	45.0
Average^c ^daily energy intake (mean MJ/d ± SD)		
9-13 yrs, males (n = 493)	9.39 ± 2.38	
9-13 yrs, females (n = 556)	8.09 ± 2.13	
14-16 yrs, males (n = 521)	11.09 ± 3.27	
14-16 yrs, females (n = 503)	8.20 ± 2.32	

^a ^Age and gender specific BMI equivalent to an adult BMI: underweight (< 18.5 kg/m^2^); normal weight (18.5-24.9 kg/m^2^); overweight (25-29.9 kg/m^2^); obese (≥ 30 kg/m^2^)

^b ^Average of four days of recording

^c ^Average of two 24 h recalls

Compared with children who had never been breastfed in infancy, those who had been breastfed for 6 or more months were significantly less likely to be overweight (adjusted OR 0.64, 95% CI 0.44-0.91) or obese (adjusted OR 0.51, 95% CI 0.29-0.90) (Table 2), after adjusting for all of the potentially predictive variables available for this analysis. There was a significant protective dose response of breastfeeding duration against overweight (P for trend = 0.002) and a similar protective trend against obesity (P for trend = 0.009) when all the potentially predictive variables were included in the model.

**Table 2:** Odds ratio of overweight^a ^and obesity^b ^among children by duration of breastfeeding in infancy (n = 2066)

	Overweight			Obesity		
	OR	(95% CI)	P for trend	OR	(95% CI)	P for trend
1. Breastfeeding duration						
0 months	1.00			1.00		
< 2 months	0.89	(0.59, 1.34)		0.84	(0.45, 1.56)	
2-< 4 months	0.66	(0.42, 1.03)		0.44	(0.20, 0.94)	
4-< 6 months	0.69	(0.46, 1.04)		0.53	(0.28, 1.02)	
≥6 months	0.54	(0.38, 0.77)	< 0.001	0.40	(0.24, 0.69)	< 0.001
2. Model 1 + age and sex of child						
0 months	1.00			1.00		
< 2 months	0.91	(0.60, 1.36)		0.85	(0.46, 1.58)	
2-< 4 months	0.67	(0.43, 1.04)		0.44	(0.20, 0.95)	
4-< 6 months	0.70	(0.47, 1.05)		0.54	(0.28, 1.03)	
≥ 6 months	0.55	(0.39, 0.77)	< 0.001	0.40	(0.24, 0.70)	< 0.001
3. Model 2 + mother's age at birth, years of secondary education and country of birth						
0 months	1.00			1.00		
< 2 months	0.94	(0.63, 1.43)		0.90	(0.48, 1.69)	
2-< 4 months	0.71	(0.46, 1.12)		0.47	(0.22, 1.03)	
4-< 6 months	0.77	(0.51, 1.13)		0.62	(0.32, 1.20)	
≥6months	0.63	(0.44, 0.99)	0.001	0.49	(0.28,0.87)	0.005
4. Model 3 + child's mean daily energy intake, physical activity, screen time and sleep duration						
0 months	1.00			1.00		
< 2 months	0.94	(0.62, 1.43)		0.88	(0.47, 1.68)	
2-< 4 months	0.70	(0.44, 1.10)		0.47	(0.22, 1.04)	
4- < 6 months	0.78	(0.52, 1.19)		0.61	(0.31, 1.20)	
≥ 6 months	0.64	(0.44, 0.91)	0.002	0.51	(0.29, 0.90)	0.009

^a ^Age and gender specific BMI equivalent to an adult BMI of ≥25 kg/m^2^

^b ^Age and gender specific BMI equivalent to an adult BMI of ≥30 kg/m^2 ^[18]

## Discussion

This study indicates that children from the 2007 Australian NCNPAS who were breastfed in infancy were less likely to be overweight or obese in later childhood. In line with past findings [6,11], there was a continuous reduction in the effect size as more explanatory variables were adjusted for in the analysis.

Children breastfed for 6 or more months were 36% less likely to be overweight and 49% less likely to be obese compared to those who were never breastfed. Furthermore, there was a protective dose-response relationship of breastfeeding duration against risk of overweight/obesity when breastfeeding duration was considered as a linear variable. This finding is supported by the findings of a meta-analysis investigating duration of breastfeeding and risk of overweight [7] whereby an inverse association existed between duration of breastfeeding and risk of overweight (regression coefficient = 0.94, 95% CI 0.89-0.98).

Evidence for the protective association of breastfeeding against overweight and obesity in later life is derived almost exclusively from observational studies. To date, PROBIT conducted by Kramer and colleagues [8] is the only (cluster) randomised controlled trial of an intervention targeted at breastfeeding initiation and duration and the effect of breastfeeding on obesity in later childhood. This trial [8] failed to find a lower prevalence of adiposity (measured as BMI and waist or hip circumference) between the intervention and the control groups suggesting that the true cause of the observed association may be one or more confounding factors not commonly controlled for in observational studies. However, this study has been criticised because it was designed to investigate the effect of the intervention on exclusive breastfeeding rather than the association of breastfeeding and obesity [20].

A common limitation of observational studies investigating the association of breastfeeding and childhood obesity is the lack of adequate adjustment for potential confounders [6]. A strength of this study was our ability to adjust for a variety of maternal sociodemographic characteristics such as age, level of education and ethnicity, which might influence both a woman's decision to breastfeed and her child's nutritional status [11], as well as measures of the study child's energy intake and expenditure (MVPA, screen time and sleep duration) reported in other studies to be associated with childhood obesity [9-13]. We were however, unable to control for maternal smoking during pregnancy and maternal BMI which have been associated with both childhood obesity [11] and duration of breastfeeding [21,22]. Another limitation of this study was the retrospective collection of breastfeeding data based on maternal recall, as recall data becomes less accurate with increased period of recall, which in this study was up to 16 years for some children [23]. Social desirability bias is a further problem associated with retrospective and extended periods of maternal recall. Mothers of older children in particular, may have been susceptible to providing "socially acceptable" responses appropriate for the current time and not the period when they were breastfeeding [23], although breastfeeding initiation and duration rates do not appear to have changed dramatically in Australia for the last 15-20 years [24]. Finally, we were unable to measure and adjust for exclusivity of breastfeeding, which is a common limitation of studies of this kind [5]. Despite these limitations, the findings of this analysis were consistent with those of previous analyses [4-7] and there appears to be general consensus that no or short breastfeeding is associated with an increased risk of overweight and obesity in later life [11].

The mechanisms that underlie the relationship between breastfeeding and childhood obesity have not been identified [25] but three possible explanations have been postulated [26]. The first explanation is that the effect is in fact spurious and created by one or more confounding factors not commonly controlled for which could be the "true" cause [26] or reflects selective reporting and/or publication bias [5]. The second explanation is that breastfed and bottle-fed infants exhibit differences in appetite control [25]. Bottle-feeding is mother-led and bottle-fed infants are usually fed on a regular basis and may be made to finish the bottle even when satiated. In contrast, breastfeeding is infant-led and there is the possibility that breastfeeding maintains the child's innate ability to regulate their energy intake and to recognise satiety signals more effectively than bottle-fed infants [25]. This theory is supported by the results of a recent study which reported that infants who were bottled-fed at 4 months of age were more likely to empty the bottle or cup in late infancy compared to infants fed directly from the breast at 4 months of age [27]. The third explanation involves the metabolic consequences of ingesting breast milk and the differences in the composition and constituents of breast milk compared with infant formula. For instance, lower serum concentration of insulin, the hormone that promotes fat storage, has been found in breastfed infants than in infants fed with infant formula [28].

## Conclusions

Breastfeeding for 6 or more months appears to be protective against later overweight and obesity in this population of Australian children. The beneficial short-term health outcomes of breastfeeding for the infant are well recognised and this study provides further observational evidence of a potential long-term health outcome and additional justification for the continued support and promotion of breastfeeding to six months and beyond.

## Competing interests

The authors declare that they have no competing interests.

## Authors' contributions

JS conceived the idea for this secondary analysis, performed part of the analysis and wrote the first and subsequent drafts of the manuscript. SN performed part of the analysis and co-wrote the first draft and reviewed subsequent drafts of the manuscript. LC was a Principle Investigator of the NCNPAS, participated in the design of this study and critically revised drafts of the paper. All authors read and approved the final version of the manuscript.

## Pre-publication history

The pre-publication history for this paper can be accessed here:

http://www.biomedcentral.com/1471-2458/12/107/prepub
